# Metabolic engineering of *Bacillus subtilis* for terpenoid production

**DOI:** 10.1007/s00253-015-6950-1

**Published:** 2015-09-15

**Authors:** Zheng Guan, Dan Xue, Ingy I. Abdallah, Linda Dijkshoorn, Rita Setroikromo, Guiyuan Lv, Wim J. Quax

**Affiliations:** 1 Department of Pharmaceutical Biology, Groningen Research Institute of Pharmacy, University of Groningen, Antonius Deusinglaan 1, Building 3215, room 917, 9713 AV Groningen, Netherlands; 2 Institute of Materia Medica, Zhejiang Chinese Medical University, Hangzhou, 310053 China

**Keywords:** Biosynthesis, Metabolic engineering, Terpenoids, *Bacillus subtilis*

## Abstract

Terpenoids are the largest group of small-molecule natural products, with more than 60,000 compounds made from isopentenyl diphosphate (IPP) and its isomer dimethylallyl diphosphate (DMAPP). As the most diverse group of small-molecule natural products, terpenoids play an important role in the pharmaceutical, food, and cosmetic industries. For decades, *Escherichia coli* (*E. coli*) and *Saccharomyces cerevisiae* (*S. cerevisiae*) were extensively studied to biosynthesize terpenoids, because they are both fully amenable to genetic modifications and have vast molecular resources. On the other hand, our literature survey (20 years) revealed that terpenoids are naturally more widespread in *Bacillales*. In the mid-1990s, an inherent methylerythritol phosphate (MEP) pathway was discovered in *Bacillus subtilis* (*B. subtilis*). Since *B. subtilis* is a generally recognized as safe (GRAS) organism and has long been used for the industrial production of proteins, attempts to biosynthesize terpenoids in this bacterium have aroused much interest in the scientific community. This review discusses metabolic engineering of *B. subtilis* for terpenoid production, and encountered challenges will be discussed. We will summarize some major advances and outline future directions for exploiting the potential of *B. subtilis* as a desired “cell factory” to produce terpenoids.

## Introduction

Nature provides an infinite treasure of complex molecules (Wilson and Danishefsky 2006) which have served as leads and scaffolds for drug discovery in the past centuries (Newman and Cragg 2007; Newman and Cragg 2012; Newman et al. 2003). Numerous reports have detailed their diverse structures and biological functions. The largest and most diverse class of small-molecule natural products is the terpenoids, also known as isoprenoids or terpenes (Köksal et al. 2011). The Dictionary of Natural Products describes approximately 359 types of terpenoids, which comprise 64,571 compounds (as of May 2015). Since these terpenoids account for ca. 24.11 % (64,571 of 267,783) of all natural products (recorded in the dictionary, http://dnp.chemnetbase.com/) and are required for biological functions in all living creatures, they indisputably play a dominant role in both the scientific community and the commercial world (Breitmaier 2006).

**Fig. 1:**
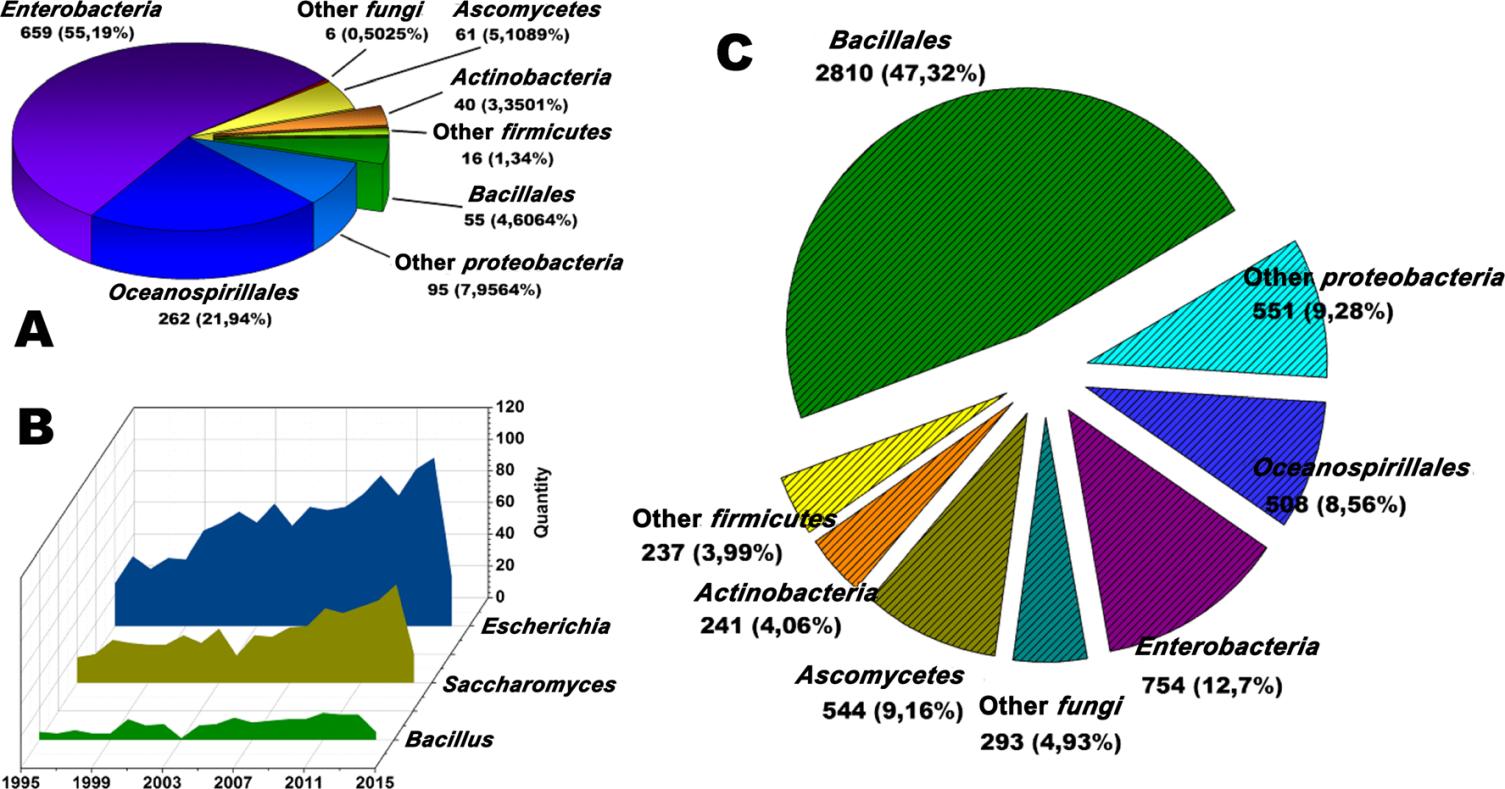
Percent of terpenoid biosynthesis related articles and terpenoid related gene reports, by source. **a** Percent of terpenoid biosynthesis related articles, by source. **b** Publication amount of terpenoid biosynthesis related articles, by year. **c** Percent of terpenoid related gene reports, by source

Along with a growing attraction for sustainable production, great interest has been expressed in biotechnological production of chemical products in general and terpenoids in particular. Since the 1990s, the interest in biosynthesizing terpenoids has skyrocketed, especially for desperately needed efficacious drugs such as artemisinin (Chang et al. 2007; Martin et al., 2003; Newman et al., 2006; Paddon et al. 2013; Ro et al. 2006; Tsuruta et al. 2009; Westfall et al. 2012) and taxol (Ajikumar et al. 2010; Jiang et al. 2012). In the past 20 years, most research has focused on using *Escherichia coli*, the host with the most advanced genetic tools, for biosynthesis of terpenoids (Fig. 1). Intensive experimentation in *Escherichia coli* (*E. coli*) has led to high yield production of some isoprenoids. However, uncertainty still looms around some aspects such as genetic engineering, characterization, reliability, quantitative strategy, and independence of biological modules (Kwok 2010). More options are needed to validate and optimize cell factories for terpenoid production. According to PubMed data, in comparison to other microorganisms, *Bacillales* (47.32 %) naturally possess more genes and proteins related to terpenoid biosynthesis pathways (Fig. 1), but surprisingly, little research effort has been devoted to the study of *Bacillales* as factories for natural products.

In the mid-1990s, it was discovered that *Bacillus subtilis*, a member of *Bacillales* that has a fast growth rate and is considered generally recognized as safe (GRAS) (FDA 1997; Schallmey et al. 2004; Widner et al. 2005), has inherent MEP pathway genes (Kuzma et al. 1995; Takahashi et al. 1998). The interest rose in *B. subtilis* as it has been used extensively for the industrial production of proteins (Westers et al. 2004; Sauer et al. 1998; Stockton and Wyss 1946). In addition, it was also reported that *Bacillus* is the highest isoprene producer among all tested microorganisms including *E. coli, Pseudomonas aeruginosa*, and *Micrococcus luteus*. The reported isoprene production rate (*B. subtilis* ATCC 6051) is 7 to 13 nmol per gram cells per hour (Kuzma et al. 1995). This high yield makes it a promising microbial host for terpenoid biosynthesis (Julsing et al. 2007; Wagner et al. 2000). Furthermore, *B. subtilis* has a wide substrate range and is able to survive under harsh conditions. Owing to its innate cellulases, it can even digest lignocellulosic materials and use the pentose sugars as its carbon source, hence decreasing the cost of biomass pretreatment (Maki et al. 2009; Ou et al. 2009). Here, we review major progress in metabolic engineering of *B. subtilis* for synthesizing terpenoids. The related pathway enzymes, genetic engineering reports, terpenoid detection methods, and their advantages and challenges will be summarized and discussed. We hope to provide a comprehensive review for exploiting the potential of *B. subtilis* as a cell factory for terpenoid production.

### Inherent terpenoid biosynthetic pathways of *B. subtilis*

**Fig. 2:**
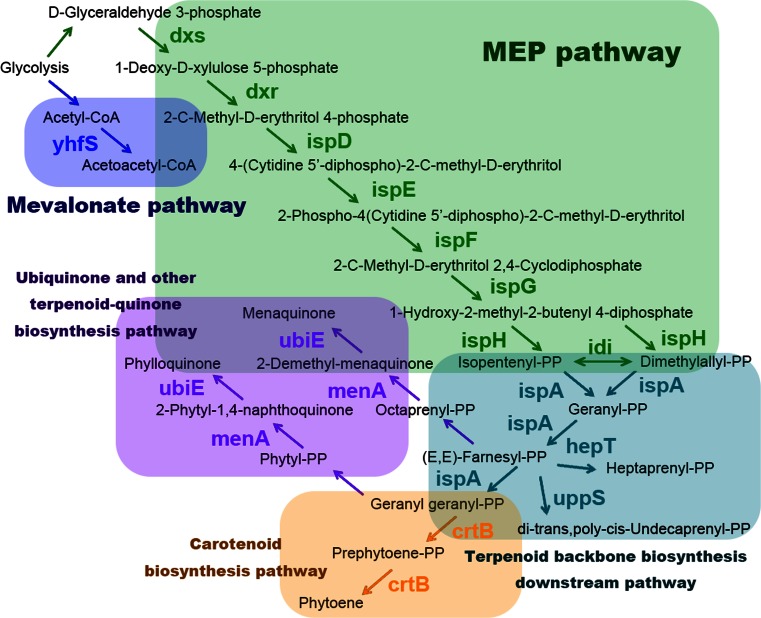
*B. subtilis* inherent terpenoid biosynthesis pathways

Terpenoids are synthesized based on isoprene (C5) units. In terpenoid biosynthetic pathways, IPP and DMAPP (C5 unit, diphosphate isoprene forms) are the basic terpenoid building blocks, generated by the Mevalonate and MEP pathways (the terpenoid backbone biosynthesis upstream pathways). The terpenoid backbone downstream pathway is responsible for biosynthesis of geranyl diphosphate (GPP), farsenyl diphosphate (FPP), and geranylgeranyl diphosphate (GGPP), which are the precursors of monoterpenoids (C10), sesquiterpenoids (C15), and diterpenoids (C20), respectively. *B. subtilis* has 15 inherent enzymes, belonging to five terpenoid biosynthesis pathways: two terpenoid backbone biosynthesis upstream pathways (the mevalonate pathway and MEP pathway), the terpenoid backbone biosynthesis downstream pathway, carotenoid biosynthesis pathway, and ubiquinone and other terpenoid-quinone biosynthesis pathway (Table 1, Fig. 2). For decades, isoprene yield has been considered the bottleneck for all terpenoid biosynthesis. Thus, to construct a cell platform which can produce and tolerate high amounts of isoprene and downstream intermediates is crucial. Since *B. subtilis* possesses all of the eight MEP pathway enzymes and can naturally produce high amounts of isoprene, it appears to be an ideal choice to utilize overexpression mutants of these enzymes to increase isoprene production.

**Table 1:** *B. subtilis* inherent terpenoid biosynthesis enzymes

Inherent pathways	EC number	Strains
Mevalonate pathway	2.3.1.9	*Bacillus subtilis* subsp. *Subtilis* 168 *Bacillus subtilis* subsp. *Subtilis* RO-NN-1 *Bacillus subtilis* subsp. *Subtilis* BSP1 *Bacillus subtilis* subsp. *Subtilis* 6051-HGW *Bacillus subtilis* subsp. *Subtilis* BAB-1 *Bacillus subtilis* subsp. *Subtilis* AG1839 *Bacillus subtilis* subsp. *Subtilis* JH642 *Bacillus subtilis* subsp. *Subtilis* OH 131.1 *Bacillus subtilis* subsp. *spizizenii* W23 *Bacillus subtilis* subsp. *spizizenii* TU-B-10 *Bacillus subtilis* subsp. *Natto* BEST195 *Bacillus subtilis* BSn5 *Bacillus subtilis* QB928 *Bacillus subtilis* XF-1 *Bacillus subtilis* PY79
MEP/DOXP pathway	2.2.1.7, 1.1.1.267, 2.7.7.60, 2.7.1.148, 4.6.1.12, 1.17.7.1, 1.17.1.2, 5.3.3.2	
Terpenoid backbone biosynthesis (downstream)	2.5.1.1, 2.5.1.10, 2.5.1.29, 2.5.1.30, 2.5.1.31	
Ubiquinone and other terpenoid-quinone biosynthesis	2.5.1.74, 2.1.1.163, 2.5.1.-	
Carotenoid biosynthesis	2.5.1.32	

• Detailed information can be found at KEGG website, http://www.kegg.jp/

• Underlined enzymes (*B. subtilis*): functional parameters can be found at the BRENDA website, http://brenda-enzymes.info/index.php

• 2.3.1.9, acetyl-CoA acetyltransferase, yhfS

• 2.2.1.7, 1-deoxy-D-xylulose-5-phosphate synthase, dxs

• 1.1.1.267, 1-deoxy-D-xylulose 5-phosphate reductoisomerase, dxr

• 2.7.7.60, 2-D-methyl-D-erythritol 4-phosphate cytidylyltransferase, ispD

• 2.7.1.148, 4-diphosphocytidyl-2-C-methyl-D-erythritol kinase, ispE

• 4.6.1.12, 2-D-methyl-D-erythritol 2,4-cyclodiphosphate synthase, ispF

• 1.17.7.1, (E)-4-hydroxy-3-methylbut-2-enyl-diphosphate synthase, ispG

• 1.17.1.2, 4-hydroxy-3-methylbut-2-enyl diphosphate reductase, ispH

• 5.3.3.2, isopentenyl-diphosphate delta-isomerase, idi

• 2.5.1.1, 2.5.1.10, 2.5.1.29, geranylgeranyl diphosphate synthase, type II, ispA

• 2.5.1.30, heptaprenyl diphosphate synthase component 2, hepT

• 2.5.1.31, undecaprenyl diphosphate synthase, uppS

• 2.5.1.74, 2.5.1.-, 1,4-dihydroxy-2-naphthoate octaprenyltransferase, menA

• 2.1.1.163, demethylmenaquinone methyltransferase, ubiE

• 2.5.1.32, phytoene synthase, crtB

However, there are few reports on the *B. subtilis* MEP pathway. Most of the MEP pathway studies are based on *E. coli*. Withers and Keasling have described the MEP pathway of *E. coli* briefly (Withers and Keasling 2007). Kuzuyama and Seto (Kuzuyama and Seto 2012) clearly illustrated the enzymes and reactions involved in the MEP pathway. Carlsen summarized MEP pathway reactions and cofactors in a table (Carlsen et al. 2013). More details can be found in Zhao’s review (Zhao et al. 2013). As the kinetics of the MEP pathway enzymes are still unknown, it is unclear which step represents the largest barrier. Thus, the lack of knowledge about the kinetic parameters of the key enzymes is the main obstacle facing metabolic engineering of the MEP pathway in *B. subtilis* to produce terpenoids. Besides that, the low number of reports about using the *B. subtilis* MEP pathway to produce terpenoids highlights the need for more research in this area.

Here, we summarize information about the MEP pathway:

1. The initial enzyme in the MEP pathway is 1-deoxy-d-xylulose-5-phosphate synthase (*dxs*), which forms 1-deoxy-d-xylulose 5-phosphate (DXP) by the condensation of d-glyceraldehyde 3-phosphate (GAP) and pyruvate. This enzyme is not only specific for the MEP pathway but also plays a role in thiamine metabolism (Sprenger et al. 1997), which shares the flux with the MEP pathway. Gene knockout results (Julsing et al. 2007) suggest that overexpressing *dxs* may result in a significant improvement in terpenoid production without notable toxicity to the host cell (Zhao et al. 2011; Zhou et al. 2013b). Previous studies in other bacteria also supported the theory that *dxs* may be the first rate-limiting step of the MEP pathway, as overexpressing *dxs* can increase isoprenoid production (Estévez et al. 2001; Kim et al. 2006; Xue and Ahring 2011). Moreover, compared to the mevalonate pathway, the theoretical mass yield of terpenoids from glucose is 30 % from DXP, 5 % higher than the yield from MVA (Rude and Schirmer 2009; Whited et al. 2010), which emphasizes the importance of *dxs* in the MEP pathway.
2. The enzymes 4-diphosphocytidyl-2-C-methyl-d-erythritol synthase (*ispD*), 4-diphosphocytidyl-2-C-methyl-d-erythritol kinase (*ispE*), and 2-C-methyl-d-erythritol 2,4-cyclodiphosphate synthase (*ispF*) are required to convert MEP to 2-C-methyl-d-erythritol 2,4-cyclodiphosphate (MECDP) (Herz et al. 2000; Kuzuyama et al. 2000a; Kuzuyama et al. 2000b; Lüttgen et al. 2000; Rohdich et al. 1999). In most organisms containing MEP pathway homologs, the genes encoding *ispD* and *ispF* are neighbors on the chromosome with the *ispE* at a distal location. They are also regarded as key enzymes in the MEP pathway (Ajikumar et al. 2010; Lu et al. 2014; Yuan et al. 2006; Zhou et al. 2013b). *IspD* and *ispF* are essential for cell survival due to their significant impact on cell wall biosynthesis and depletion (Campbell and Brown 2002). *IspE* has also been identified as crucial for survival of pathogenic bacteria and essential in *Mycobacterium smegmatis* (Eoh et al. 2009).
3. The most controversial enzymes in the MEP pathway are 1-deoxy-d-xylulose-5-phosphate reductoisomerase (*dxr*) and isopentenyl-diphosphate delta-isomerase (*idi*). Some researchers consider them as key enzymes in the MEP pathway (Berthelot et al. 2012; Soliman et al. 2011; Sun et al. 1998; Xue et al. 2015), while others find that they are not essential, at least in some cases (Fox and Poulter 2005; Lagarde et al. 2000; Sangari et al. 2010; Xue and Ahring 2011; Zhao et al. 2011). As far as we know now, there are two families of *idi*, *B. subtilis* possesses type 2 *idi*, which was considered as a nonessential enzyme in the *bacillus* MEP pathway (Julsing et al. 2007; Takagi et al. 2004).
4. Other important enzymes in the MEP pathway are (E)-4-hydroxy-3-methylbut-2-enyl-diphosphate synthase (*ispG*) and 4-hydroxy-3-methylbut-2-enyl diphosphate reductase (*ispH*), but their catalytic mechanisms are still unclear (Zhao et al. 2013). The enzyme *ispH* catalyzes the 2H^+^∕2e^−^ reduction of hydroxy-2-methyl-2-butenyl-4-diphosphate (HMBDP) producing an approximately 5:1 mixture of IPP and DMAPP in return (Wang et al. 2010). This enzyme and *ispG* are deemed essential enzymes for cell survival (Liu et al. 2012; Rohmer 2008). It has been reported that *ispG* can effectively reduce the efflux of methylerythritol cyclodiphosphate (MECDP), resulting in a significant increase in downstream terpenoid production (Zhou et al. 2012). Additional information on the bio-organometallic chemistry of *ispG* and *ispH* can be found in Wang’s review (Wang and Oldfield 2014).

### Genetic engineering of *B. subtilis*

Most of the knowledge about the MEP pathway was obtained from research in *E. coli* and other bacteria. Therefore, research into the progress of genetic engineering of MEP pathway enzymes in *B. subtilis* can provide more direct support for utilizing *B. subtilis* as a microbial host for terpenoid biosynthesis.

Wagner first described the phases of isoprene formation during growth and sporulation of *B. subtilis* (Wagner et al. 1999). They found that isoprene formation is linked to glucose catabolism, acetoin catabolism, and sporulation. One possible mechanism is that isoprene is a metabolic overflow metabolite released when flow of carbon to higher isoprenoids is restricted. This phenomenon can be illustrated as follows: (a) when cells are rapidly metabolizing the available carbon sources, isoprene is released; (b) when less carbon is available during transitions in carbon assimilation pathways, isoprene production declines; and (c) when cell growth ceases and spore formation is initiated, production of isoprene continues. In 2000, it was confirmed that isoprene is a product of the MEP pathway in *B. subtilis* (Wagner et al. 2000). It was also reported that isoprene release might be used as a barometer of central carbon flux changes during the growth of *Bacillus* strains (Shirk et al. 2002). Besides that, the activity of isoprene synthase (ISPS) was studied by using permeabilized cells. When grown in a bioreactor, *B. subtilis* cells released isoprene in parallel with the ISPS activity (Sivy et al. 2002).

In order to gain more insight into the MEP pathway of *B. subtilis*, conditional knockouts of the MEP pathway genes of *B. subtilis* were constructed, then the amount of emitted isoprene was analyzed. The results show that the emission of isoprene is severely decreased without the genes encoding *dxs*, *ispD*, *ispF*, or *ispH*, indicating their importance in the MEP pathway. In addition, *idi* has been proven not to be essential for the *B. subtilis* MEP pathway (Julsing et al. 2007). Xue and Ahring first tried to enhance isoprene production by modifying the MEP pathway in *B. subtilis*. They overexpressed the *dxs* and *dxr* genes. The strain that overexpressed *dxs* showed a 40 % increase in isoprene yield compared to the wild-type strain, whereas in the *dxr* overexpression strain, the isoprene level was unchanged. Furthermore, they studied the effect of external factors and suggested that 1 % ethanol inhibits isoprene production, but the stress factors heat (48 °C), salt (0.3 M), and H_2_O_2_ (0.005 %) can induce the production of isoprene. In addition, they found that these effects are independent of SigB, which is the general stress-responsive alternative sigma factor of *B. subtilis* (Xue and Ahring 2011). Hess et al. co-regulated the terpenoid pathway genes in *B. subtilis*. Transcriptomics results showed that the expression levels of *dxs* and *ispD* are positively correlated with isoprene production, while on the other hand, the expression levels of *ispH*, *ispF*, *ispE*, and *dxr* are inversely correlated with isoprene production. Moreover, their results supported Xue’s conclusions about the effect of external factors (Hess et al. 2013).

In 2009, Yoshida et al. first successfully transcribed and transfected *crtM* and *crtN* genes into *B. subtilis* to direct the carbon flux from the MEP pathway to C_30_ carotenoid biosynthesis and successfully produced 4,4′-diapolycopene and 4,4′-diaponeurosporene (Yoshida et al. 2009). Thereafter, Maeda reported a method to produce glycosylated C_30_ carotenoic acid by introducing *Staphylococcus aureus* (*S. aureus*) *crtP* and *crtQ* genes into *B. subtilis*, together with *crtM* and *crtN* (Maeda 2012). Later, Zhou overexpressed *dxs* and *idi* genes along with introducing *ads* (*ads* encodes the synthase which cyclizes farnesyl diphosphate into amorphadiene) in *B. subtilis* and got the highest yield of amorphadiene (∼20 mg/L) at shake-flask scale. They thought that the lack of genetic tools for fine-tuning the expression of multiple genes is the bottleneck in production of terpenoids in *B. subtilis*. So they modified *B. subtilis* genes by using a two-promoter system to independently control the expression levels of two gene cassettes (Zhou et al. 2013a). After that, Xue et al. systematically studied the *B. subtilis* MEP pathway enzymes (Xue et al. 2015). A series of synthetic operons expressing MEP pathway genes were analyzed by using the level of C30 carotenoid production as a measure of the effect of those modulations. All of the overexpressed gene constructs showed higher production of carotenoids compared to wild type. *Dxs* and *dxr* (8-fold and 9.2-fold increase in carotenoid production) have been validated as the most productive part of the MEP pathway genes in this study.

Other reports are related to C_35_ terpenoids and their enzymes, which were found in *B. subtilis*, like heterodimeric enzyme, heptaprenyl diphosphate synthase (HepS and HepT), and tetraprenyl-β-curcumene synthase (YtpB), which are responsible for forming long prenyl diphosphate chains (C_35_) (Sato et al. 2011). As Heider noted in his review, *B. subtilis* has not yet been a major focus to produce carotenoids (Heider et al. 2014). Furthermore, we cannot find other research about terpenoid biosynthesis in *B. subtilis*. Since *B. subtilis* possesses many advantages as mentioned above in the introduction section, biosynthesis of terpenoids via the *B. subtilis* MEP pathway could be both an opportunity and a challenge.

### Detection and metabolomics methods for engineering terpenoid pathway

As is known, most metabolic engineering work is improved by using a combination of random and targeted approaches. Mariët and Renger (Wilson and Danishefsky 2006) pointed out that the selection of these targets has depended at best on expert knowledge but to a great extent also on “educated guesses” and “gut feeling.” Consequently, time and money are wasted on irrelevant targets or only a minor improvement result. Along with the development of systems biology, metabolomics, a technology that includes non-targeted, holistic metabolite analysis of the cellular and/or environmental changes combined with multivariate data analysis tools is being increasingly used to replace empirical approaches for targeted natural product biosynthesis (Newman et al. 2006; Paddon et al. 2013). Gregory’s group (Ajikumar et al. 2010) has used metabolomics analysis of their previous strains, leading them to identify a noticeable metabolite by-product that inversely correlated with taxadiene accretion. This hint helped them to achieve approximately 1 g per liter taxadiene from *E. coli*.

**Scheme 1:**
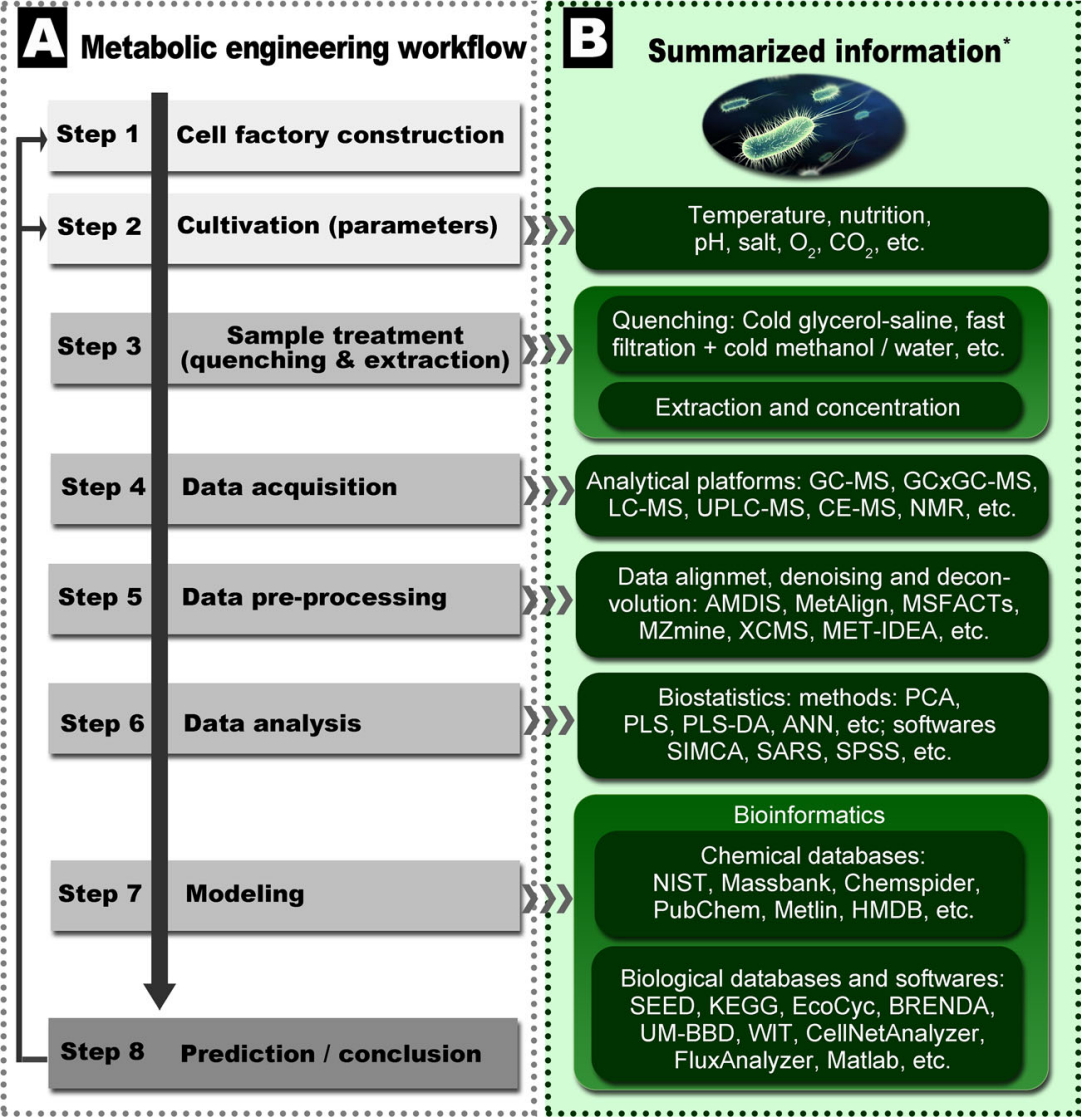
Flowchart and resources for terpenoid microbial metabolomics study. **a** Microbial metabolic engineering workflow. **b** Related information of each step for microbial metabolic engineering. * Selected resources: 1. MS data of *B. subtilis* metabolites (Coulier et al. 2006; Koek et al. 2006; Soga et al. 2003). 2. The metabolomics standards initiative (Fiehn et al. 2007). 3. Microbial metabolomics study examples for terpenoid biosynthesis (Paddon and Keasling 2014; Zhou et al. 2012). 4. Databases, software packages, and protocols (Thiele and Palsson 2010) and http://omictools.com/. 5. Genome-scale data of reconstructed *B. subtilis* metabolic net (impact of single-gene deletions on growth in *B. subtilis*) (Oh et al. 2007). 6. Comparative microbial metabolomics study of *E. coli*, *B. subtilis*, and *S. cerevisiae* (van der Werf et al. 2007). 7. The complete genome sequence of *B. subtilis* (Kunst et al. 1997). 8. Constraint-based modeling methods (Bordbar et al. 2014). 9. Software applications for flux balance analysis (including a software comparative list) (Lakshmanan et al. 2012). 10. Sample treatment methods (Jia et al. 2004; Larsson and Törnkvist 1996; Maharjan and Ferenci 2003; van der Werf et al. 2007; Villas-Bôas and Bruheim 2007)

Because the research on the *Bacillus* MEP pathway is still at an early stage, it is urgent to develop guidelines for unbiased selection of the best rational design approach to engineering the terpenoid. The newest developments of metabolomics, meta-omics, computer, and mathematic sciences offer more options for not only unbiased selection and ranking methods but also high-throughput and more precise prediction models that enable a mechanistic description of microbial metabolic pathways (Breitmaier 2006; Martin et al., 2003). Scheme 1 summarizes the workflow, essential reports, and resources for the study of terpenoid microbial metabolomics.

To observe and optimize the terpenoid biosynthesis pathways, detection methods are also crucial. The techniques that are currently employed in the study of microbially produced terpenoids are usually gas chromatography-mass spectrometry (GC-MS) and liquid chromatography-MS (LC-MS). Other techniques such as nuclear magnetic resonance (NMR) (Hecht et al. 2001) and Raman spectroscopic analysis (de Oliveira et al. 2010) are also used in terpenoid analysis, although compared with MS-based coupling techniques, they are less sensitive and/or reliable. Most likely, the currently existing methods for the quantitative determination of terpenoids in bacteria are sufficient. There are numerous articles about quantifying and identifying terpenoids (esp. carotenoids, see Foppen’s tables (Foppen 1971)) in plants, microorganisms, and other organisms. Most of these methods can be applied in *B. subtilis*.

In 1995, Kuzma discovered that *B. subtilis* can produce isoprene efficiently (Kuzma et al. 1995). This Colorado research group focused on the isoprene biosynthesis mechanism in *B. subtilis*. They used GC, GC-MS, HPLC, ^13^C, and ^2^H labeling methods, non-radioactive methods, and online chemical-ionization mass spectrometry (CIMS) to measure isoprene and MEP pathway metabolites (Custer et al. 2003; Fisher et al. 2001; Kuzma et al. 1995; Shirk et al. 2002; Wagner et al. 2000; Wagner et al. 1999). Table 2 summarizes their methods, as well as more recent methods to detect and analyze *B. subtilis* terpenoid metabolites.

**Table 2:** Detection and analysis reports of *B. subtilis* terpenoid pathway metabolites

Method	Compound	Characteristic	Reference
GC-MS	Isoprene	Rt = 16.5 min, *m*/*z* 39, 53, 67	Kuzma et al. 1995
GC	Isoprene		Wagner et al. 1999
GC-MS (^13^C, ^2^H labeling)	Isoprene	Common substrate *m*/*z* 39, 53, 67; Substrate: U-[^13^C_6_] glucose *m*/*z* 42, 57, 72; 1-[^13^C] pyruvate *m*/*z* 40, 54, 68; 2-[^13^C] pyruvate *m*/*z* 40, 55, 69; 3-[^13^C] pyruvate *m*/*z* 40, 54, 69.	Wagner et al. 2000
GC	DMAPP		Fisher et al. 2001
GC	Isoprene	Rt = 2.6 min	Shirk et al. 2002
HPLC	Acetoin	Rt = 5.5 min, 354 nm	
Kits	Glucose		
Kits	Lactic, pyruvic acids		
CIMS		(M + H)^+^(H_2_O)_*n*_:	Custer et al. 2003
	Acetaldehyde	*m*/*z* 63 (*n* = 1)	
	Acetoin	*m*/*z* 89 (*n* = 0)	
	Acetone	*m*/*z* 77 (*n* = 1)	
	2,3-Butanediol	*m*/*z* 91 (*n* = 0)	
	Butanol	*m*/*z* 111 (*n* = 2)	
	2-Butanone	*m*/*z* 91 (*n* = 1)	
	Butyraldehyde	*m*/*z* 91 (*n* = 1)	
	Butyl acetate	*m*/*z* 135 (*n* = 1)	
	Diacetyl	*m*/*z* 123 (*n* = 2)	
	Dimethyl sulfide	*m*/*z* 63 (*n* = 0)	
	Ethanol	*m*/*z* 83 (*n* = 2)	
	Ethyl acetate	*m*/*z* 107 (*n* = 1)	
	Isoamyl alcohol	*m*/*z* 107 (*n* = 1)	
	Isoprene	*m*/*z* 69 (*n* = 0)	
GC	Isoprene		Julsing et al. 2007
HPLC	4,4′-Diapolycopene	Rt = 26.8 min, Absorption: 293, 443, 472, 501 nm	Yoshida et al. 2009
	4,4′-Diaponeurosporene	Rt = 28.9 min, Absorption: 266, 415, 439, 469 nm	
MALDI-TOF MS	4,4′-Diapolycopene	*m*/*z* 399.9	
	4,4′-Diaponeurosporene	*m*/*z* 401.9	
GC-MS	Isoprene	Rt = 1.9 min	Xue and Ahring 2011
HPLC	Glycosyl 4,4′-diaponeurosporenoate	Rt = 10.0 min, Absorption: 282, 469 nm	Barredo 2012
	4,4′-Diapolycopene	Absorption: 293, 443, 472, 501 nm	
	4,4′-Diaponeurosporene	Rt = 14.4 min, Absorption: 265, 414, 441, 469 nm	
UPLC-MS	DXP	Rt = 5.6 min, *m*/*z* 213.0170	Tsuruta et al. 2009; Zhou et al. 2012; Zhou et al. 2013a
	MEP	Rt = 5.2 min, *m*/*z* 215.0330	
	CDP-ME	Rt = 6.2 min, *m*/*z* 520.0730	
	CDP-MEP	Rt = 7.3 min, *m*/*z* 600.0390	
	MEC	Rt = 6.6 min, *m*/*z* 276.9884	
	HMBPP	Rt = 7.0 min, *m*/*z* 260.9920	
GC-MS	*Trans-*Caryophyllene	Rt = 3.4 min, *m*/*z* 189, 204	
	Amorpha-4,11-diene	Rt = 3.5 min, *m*/*z* 189, 204	
LC-Fourier transform MS	(Untargeted metabolomics study)		Cho et al. 2014

As is the case for biosynthesis of different chemical compounds, genetic modification often leads to dead ends. The difficulties in metabolic engineering of bacteria for terpenoid production normally are not terpenoid detection but problems in the complex metabolic net (Baidoo and Keasling 2013). Although the latest reports (Zhou et al. 2012; Zhou et al. 2013a) describe a promising method that can simultaneously detect MEP pathway intermediates, the repeatability is not as good for CDP-MEP as for the other intermediates, especially when the amount of CDP-MEP in bacteria is very low (summarized MS information of MEP pathway metabolites can be found in Table 3). In addition, even if the reported methods are sufficient to analyze all the MEP pathway intermediates, it is still difficult to predict and identify the unknown mechanisms for improving terpenoid production and other relevant compounds due to the fact that all of the MEP pathway enzymes are also involved in other metabolic activities (http://www.kegg.jp/). Cho’s untargeted metabolomics study (Cho et al. 2014) may have pointed out a direction that can help solve some of these problems, whereas few untargeted metabolomics research for *B. subtilis* metabolic pathway study can be found online. As mentioned above, integrated metabolomics studies and constraint-based models might orient future study for biosynthesis of terpenoids (see Scheme 1). The current state of analysis methods, which can be integrated into metabolomics researches and be used in terpenoid biosynthesis studies, raises questions about the following issues: (1) detailed preparation work such as reproducible growth of *B. subtilis*, sampling, and quenching methods, which can be used in metabolomics studies to elucidate the mechanisms of the MEP pathway; (2) extraction methods that maintain the original structure of intermediates and subsequently allow the identification of those compounds and their accurate quantification; (3) extraction coupled quantification methods that can be used to quantify minor components from small-scale bacterial cultures to reduce the workload; and (4) data pre-processing, biostatistics, and bioinformatics methods for big data analysis, integration, and modeling that can reflect the cell bio-net, narrow the research scope, target the key products, genes, and enzymes, and finally lead us to further improvements.

**Table 3:** MS information of *B. subtilis* inherent terpenoid pathway intermediates

Compound	Formula	Mass	ESI-Q-TOF		
			Mode	CE (V)	m/z
G3P	C_3_H_7_O_6_P	169.9980	+	40	80.9730, 62.9631, 98.9823, 45.0347
DXP	C_5_H_11_O_7_P	214.0242	−	0	213.0167, 96.9695, 138.9795,78.9592
MEP	C_5_H_13_O_7_P	216.0399			
CDP-ME	C_14_H_25_N_3_O_14_P_2_	521.0812			
CDP-ME2P	C_14_H_26_N_3_O_17_P_3_	601.0475			
MECDP	C_5_H_12_O_9_P_2_	277.9957	+	40	98.9830, 83.0480, 55.0538, 65.0394, 80.9733, 43.0536
HMBDP	C_5_H_12_O_8_P_2_	262.0007			
IPP	C_5_H_12_O_7_P_2_	246.0058	−	0	244.9979, 78.9591
DMAPP	C_5_H_12_O_7_P_2_	246.0058	−	0	244.9979, 78.9592
GPP	C_10_H_20_O_7_P_2_	314.0684	−	0	313.0629, 78.9593
FPP	C_15_H_28_O_7_P_2_	382.1310			
GGPP	C_20_H_36_O_7_P_2_	450.1936			
PPDP	C_40_H_68_O_7_P_2_	722.4440			
Phytoene	C_40_H_64_	544.5008			
HepPP	C_35_H_60_O_7_P_2_	654.3814			
UDPP	C_55_H_92_O_7_P_2_	926.6318			
PDP	C_20_H_42_O_7_P_2_	456.2406			
OPP	C_40_H_68_O_7_P_2_	722.4440			
2-Phytyl-1,4-naphthoquinone	C_30_H_44_O_2_	436.3341			
2-Demethylmenaquinone	C_50_H_70_O_2_	702.5376			
Phylloquinone	C_31_H_46_O_2_	450.3498	+	40	187.0749, 57.0703, 43.0550, 71.0856, 171.0799, 199.0758, 105.0326, 157.0650
Menaquinone	C_41_H_56_O_2_	580.4280			

• Data sources: http://www.hmdb.ca/, http://www.massbank.jp/index.html?lang=en, http://www.chemspider.com/, https://metlin.scripps.edu/index.php, http://pubchem.ncbi.nlm.nih.gov/

• *G3P* d-glyceraldehyde 3-phosphate

• *DXP* deoxy-d-xylulose 5-phosphate

• *MEP* 2-C-methyl-d-erythritol 4-phosphate

• *CDP-ME* 4-(cytidine 5′-diphospho)-2-C-methyl-d-erythritol

• *CDP-ME2P* phospho-4(Cytidine 5′-diphospho)-2-C-methyl-d-erythritol

• *MECDP* 2-C-methyl-d-erythritol 2,4-Cyclodiphosphate

• *HMBDP* hydroxy-2-methyl-2-butenyl 4-diphosphate

• *IPP* isopentenyl-PP

• *DMAPP* dimethylallyl-PP

• *GPP* geranyl-PP

• *FPP* (E,E)-farnesyl-PP

• *GGPP* geranylgeranyl-PP

• *PPDP* prephytoene-PP.

• *HepPP* heptaprenyl-PP

• *UDPP* di-trans, poly-cis-undecaprenyl-PP

• *PDP* phytyl-PP

• *OPP* octaprenyl-PP

### Summary

*B. subtilis* offers new opportunities and good prospects for terpenoid biosynthesis. This review provides a brief account of metabolic engineering of *B. subtilis* for terpenoid production, summarizing our understanding of *B. subtilis*, the MEP pathway, and related techniques. While the mevalonate pathway and terpenoid biosynthesis in *E. coli* have been studied for decades, research on the *Bacillus* MEP pathway is still at an early stage. That is why, at this point, there is no sufficient data on *Bacillus* yield to make a fair comparison with published yields of terpenoids in *E. coli* and other cell factories. However, theoretically, *B. subtilis* has the potential to be optimized as a high-yield-producing cell factory. The advantages of studying terpenoid biosynthesis in *B. subtilis* include (1) its fast growth rate and ability to survive under harsh conditions, (2) its GRAS status, (3) its wide substrate range and inherent MEP pathway genes, (4) the fact that it is a naturally high isoprene producer, (5) its clear genetic background, abundant genetic tools, and (6) its innate cellulases, which can digest lignocellulosic materials and use the breakdown products as its carbon source, which would decrease large-scale production costs. Still, *B. subtilis* share some of the features of other gram-positive bacteria like plasmid instability. Also, there are some *B. subtilis*-specific engineering challenges that need to be explored. The catalytic mechanisms of two MEP pathway enzymes (IspG, IspH) in *B. subtilis* are unclear yet. The importance of DXR and IDI in the MEP pathway is controversial. DXS has been generally regarded as the essential rate-limiting enzyme, but even the functional parameters of DXS in *B. subtilis* have not yet been reported. Many questions regarding the mechanism of the MEP pathway, the interactions of related enzymes and metabolites, and the kinetic parameters of MEP pathway enzymes in *B. subtilis* remain unanswered. Obviously, the organism is promising and the questions are fascinating. There is thus significant reason for detailed investigations of terpenoid biosynthesis via the *B. subtilis* MEP pathway, particularly in metabolic engineering where there is not yet sufficient knowledge about the precise mechanisms or the effects of co-regulation of the enzymes.

## References

[CR1] Ajikumar PK, Xiao W-H, Tyo KE, Wang Y, Simeon F, Leonard E, Mucha O, Phon TH, Pfeifer B, Stephanopoulos G (2010). Isoprenoid pathway optimization for Taxol precursor overproduction in *Escherichia coli*. Science (New York, NY).

[CR2] Baidoo EE, Keasling JD (2013). Microbial metabolomics: welcome to the real world!. Metabolomics.

[CR3] Barredo J-L (2012). Microbial carotenoids from bacteria and microalgae. Methods Mol Biol.

[CR4] Berthelot K, Estevez Y, Deffieux A, Peruch F (2012). Isopentenyl diphosphate isomerase: a checkpoint to isoprenoid biosynthesis. Biochimie.

[CR5] Bordbar A, Monk JM, King ZA, Palsson BO (2014). Constraint-based models predict metabolic and associated cellular functions. Nat Rev Genet.

[CR6] Breitmaier E (2006) Terpenes: flavors, fragrances, pharmaca, pheromones. John Wiley & Sons

[CR7] Campbell TL, Brown ED (2002) Characterization of the depletion of 2-*C*-methyl-D-erythritol-2,4-cyclodiphosphate synthase in *Escherichia coli* and *Bacillus subtilis*. J Bacteriol 184(20):5609–5618 10.1128/JB.184.20.5609-5618.2002 PMC139617 12270818

[CR8] Carlsen S, Ajikumar PK, Formenti LR, Zhou K, Phon TH, Nielsen ML, Lantz AE, Kielland-Brandt MC, Stephanopoulos G (2013) Heterologous expression and characterization of bacterial 2-*C*-methyl-D-erythritol-4-phosphate pathway in *Saccharomyces cerevisiae*. Appl Microbiol Biotechnol 97(13):5753–5769. doi:10.1007/s00253-013-4877-y 10.1007/s00253-013-4877-y 23636690

[CR9] Chang MCY, Eachus RA, Trieu W, Ro D-K, Keasling JD (2007). Engineering *Escherichia coli* for production of functionalized terpenoids using plant P450s. Nat Chem Biol.

[CR10] Cho K, Evans BS, Wood BM, Kumar R, Erb TJ, Warlick BP, Gerlt JA, Sweedler JV (2014) Integration of untargeted metabolomics with transcriptomics reveals active metabolic pathways. Metabolomics 2014(August). doi:10.1007/s11306-014-0713-3 10.1007/s11306-014-0713-3 PMC4334135 25705145

[CR11] Coulier L, Bas R, Jespersen S, Verheij E, van der Werf MJ, Hankemeier T (2006). Simultaneous quantitative analysis of metabolites using ion-pair liquid chromatography-electrospray ionization mass spectrometry. Anal Chem.

[CR12] Custer TG, Wagner WP, Kato S, Bierbaum VM, Fall R (2003). Potential of on-line CIMS for bioprocess monitoring. Biotechnol Prog.

[CR13] de Oliveira VE, Castro HV, Edwards HG, de Oliveira LFC (2010). Carotenes and carotenoids in natural biological samples: a Raman spectroscopic analysis. J Raman Spectrosc.

[CR14] Eoh H, Narayanasamy P, Brown AC, Parish T, Brennan PJ, Crick DC (2009) Expression and characterization of soluble 4-diphosphocytidyl-2-*C*-methyl-D-erythritol kinase from bacterial pathogens. Chem Biol 16(12):1230–1239 10.1016/j.chembiol.2009.10.014 PMC4020808 20064433

[CR15] Estévez JM, Cantero A, Reindl A, Reichler S, León P (2001). 1-Deoxy-D-xylulose-5-phosphate synthase, a limiting enzyme for plastidic isoprenoid biosynthesis in plants. J Biol Chem.

[CR16] FDA (1997). FDA proposed simplified. GRAS notification system - 62 FR 18937.

[CR17] Fiehn O, Robertson D, Griffin J, van der Werf M, Nikolau B, Morrison N, Sumner LW, Goodacre R, Hardy NW, Taylor C (2007). The metabolomics standards initiative (MSI). Metabolomics.

[CR18] Fisher AJ, Rosenstiel TN, Shirk MC, Fall R (2001). Nonradioactive assay for cellular dimethylallyl diphosphate. Anal Biochem.

[CR19] Foppen FH (1971). Tables for the identification of carotenoid pigments. Chromatogr Rev.

[CR20] Fox DT, Poulter CD (2005) Mechanistic studies with 2-*C*-methyl-D-erythritol 4-phosphate synthase from *Escherichia coli*. Biochemistry 44(23):8360–8368. doi:10.1021/bi047312p 10.1021/bi047312p 15938625

[CR21] Hecht S, Eisenreich W, Adam P, Amslinger S, Kis K, Bacher A, Arigoni D, Rohdich F (2001). Studies on the nonmevalonate pathway to terpenes: the role of the GcpE (IspG) protein. Proc Natl Acad Sci.

[CR22] Heider SAE, Peters-Wendisch P, Wendisch VF, Beekwilder J, Brautaset T (2014). Metabolic engineering for the microbial production of carotenoids and related products with a focus on the rare C_50_ carotenoids. Appl Microbiol Biotechnol.

[CR23] Herz S, Wungsintaweekul J, Schuhr CA, Hecht S, Lüttgen H, Sagner S, Fellermeier M, Eisenreich W, Zenk MH, Bacher A (2000) Biosynthesis of terpenoids: YgbB protein converts 4-diphosphocytidyl-2*C*-methyl-D-erythritol 2-phosphate to 2*C*-methyl-D-erythritol 2, 4-cyclodiphosphate. Proc Natl Acad Sci 97(6):2486–2490 10.1073/pnas.040554697 PMC15955 10694574

[CR24] Hess BM, Xue J, Markillie LM, Taylor RC, Wiley HS, Ahring BK, Linggi B (2013). Coregulation of terpenoid pathway genes and prediction of isoprene production in using transcriptomics. PLoS One.

[CR25] Jia L, Liu B-F, Terabe S, Nishioka T (2004). Two-dimensional separation method for analysis of *Bacillus subtilis* metabolites via hyphenation of micro-liquid chromatography and capillary electrophoresis. Anal Chem.

[CR26] Jiang M, Stephanopoulos G, Pfeifer BA (2012). Downstream reactions and engineering in the microbially reconstituted pathway for Taxol. Appl Microbiol Biotechnol.

[CR27] Julsing MK, Rijpkema M, Woerdenbag HJ, Quax WJ, Kayser O (2007). Functional analysis of genes involved in the biosynthesis of isoprene in *Bacillus subtilis*. Appl Microbiol Biotechnol.

[CR28] Kim S-J, Kim M-D, Choi J-H, Kim S-Y, Ryu Y-W, Seo J-H (2006). Amplification of 1-deoxy-D-xyluose 5-phosphate (DXP) synthase level increases coenzyme Q(10) production in recombinant *Escherichia coli*. Appl Microbiol Biotechnol.

[CR29] Koek MM, Muilwijk B, van der Werf MJ, Hankemeier T (2006). Microbial metabolomics with gas chromatography/mass spectrometry. Anal Chem.

[CR30] Köksal M, Hu H, Coates RM, Peters RJ, Christianson DW (2011). Structure and mechanism of the diterpene cyclase ent-copalyl diphosphate synthase. Nat Chem Biol.

[CR31] Kunst F, Ogasawara N, Moszer I, Albertini A, Alloni G, Azevedo V, Bertero M, Bessieres P, Bolotin A, Borchert S (1997). The complete genome sequence of the gram-positive bacterium *Bacillus subtilis*. Nature.

[CR32] Kuzma J, Nemecek-Marshall M, Pollock WH, Fall R (1995). Bacteria produce the volatile hydrocarbon isoprene. Curr Microbiol.

[CR33] Kuzuyama T, Seto H (2012). Two distinct pathways for essential metabolic precursors for isoprenoid biosynthesis. Proc Japan Acad Ser B-Phys Biol.

[CR34] Kuzuyama T, Takagi M, Kaneda K, Dairi T, Seto H (2000a) Formation of 4-(cytidine 5′-diphospho)-2-*C*-methyl-D-erythritol from 2-*C*-methyl-D-erythritol 4-phosphate by 2-*C*-methyl-D-erythritol 4-phosphate cytidylyltransferase, a new enzyme in the nonmevalonate pathway. Tetrahedron Lett 41(5):703–706

[CR35] Kuzuyama T, Takagi M, Kaneda K, Watanabe H, Dairi T, Seto H (2000b) Studies on the nonmevalonate pathway: conversion of 4-(cytidine 5′-diphospho)-2-*C*-methyl-D-erythritol to its 2-phospho derivative by 4-(cytidine 5′-diphospho)-2-*C*-methyl-D-erythritol kinase. Tetrahedron Lett 41(16):2925–2928

[CR36] Kwok R (2010). Five hard truths for synthetic biology. Nat News.

[CR37] Lagarde D, Beuf L, Vermaas W (2000). Increased production of zeaxanthin and other pigments by application of genetic engineering techniques to *Synechocystis* sp. strain PCC 6803. Appl Environ Microbiol.

[CR38] Lakshmanan M, Koh G, Chung BK, Lee D-Y (2012) Software applications for flux balance analysis. Briefings in bioinformatics:bbs069 10.1093/bib/bbs069 23131418

[CR39] Larsson G, Törnkvist M (1996). Rapid sampling, cell inactivation and evaluation of low extracellular glucose concentrations during fed-batch cultivation. J Biotechnol.

[CR40] Liu Y-L, Guerra F, Wang K, Wang W, Li J, Huang C, Zhu W, Houlihan K, Li Z, Zhang Y, Nair SK, Oldfield E (2012). Structure, function and inhibition of the two- and three-domain 4Fe-4S IspG proteins. Proc Natl Acad Sci U S A.

[CR41] Lu W, Ye L, Xu H, Xie W, Gu J, Yu H (2014) Enhanced production of coenzyme Q(10) by self-regulating the engineered MEP pathway in *Rhodobacter sphaeroides*. Biotechnol Bioeng 111(4):761–769. doi:10.1002/bit.25130 10.1002/bit.25130 24122603

[CR42] Lüttgen H, Rohdich F, Herz S, Wungsintaweekul J, Hecht S, Schuhr CA, Fellermeier M, Sagner S, Zenk MH, Bacher A (2000) Biosynthesis of terpenoids: YchB protein of *Escherichia coli* phosphorylates the 2-hydroxy group of 4-diphosphocytidyl-2*C*-methyl-D-erythritol. Proc Natl Acad Sci 97(3):1062–1067 10.1073/pnas.97.3.1062 PMC15522 10655484

[CR43] Maeda I (2012) Genetic modification in *Bacillus subtilis* for production of C_30_ carotenoids. Methods in molecular biology (Clifton, NJ) 892:197–205 doi:10.1007/978–1–61779–879-5_11 10.1007/978-1-61779-879-5_11 22623304

[CR44] Maharjan RP, Ferenci T (2003). Global metabolite analysis: the influence of extraction methodology on metabolome profiles of *Escherichia coli*. Anal Biochem.

[CR45] Maki M, Leung KT, Qin W (2009). The prospects of cellulase-producing bacteria for the bioconversion of lignocellulosic biomass. Int J Biol Sci.

[CR46] Martin VJ, Pitera DJ, Withers ST, Newman JD, Keasling JD (2003). Engineering a mevalonate pathway in *Escherichia coli* for production of terpenoids. Nat Biotechnol.

[CR47] Newman DJ, Cragg GM (2007) Natural products as sources of new drugs over the last 25 years. J Nat Prod 70(3):461–477 10.1021/np068054v 17309302

[CR48] Newman DJ, Cragg GM (2012). Natural products as sources of new drugs over the 30 years from 1981 to 2010. J Nat Prod.

[CR49] Newman DJ, Cragg GM, Snader KM (2003). Natural products as sources of new drugs over the period 1981–2002. J Nat Prod.

[CR50] Newman JD, Marshall J, Chang M, Nowroozi F, Paradise E, Pitera D, Newman KL, Keasling JD (2006). High-level production of amorpha-4, 11-diene in a two-phase partitioning bioreactor of metabolically engineered *Escherichia coli*. Biotechnol Bioeng.

[CR51] Oh Y-K, Palsson BO, Park SM, Schilling CH, Mahadevan R (2007). Genome-scale reconstruction of metabolic network in *Bacillus subtilis* based on high-throughput phenotyping and gene essentiality data. J Biol Chem.

[CR52] Ou MS, Mohammed N, Ingram L, Shanmugam K (2009). Thermophilic *Bacillus coagulans* requires less cellulases for simultaneous saccharification and fermentation of cellulose to products than mesophilic microbial biocatalysts. Appl Biochem Biotechnol.

[CR53] Paddon C, Westfall P, Pitera D, Benjamin K, Fisher K, McPhee D, Leavell M, Tai A, Main A, Eng D (2013). High-level semi-synthetic production of the potent antimalarial artemisinin. Nature.

[CR54] Paddon CJ, Keasling JD (2014). Semi-synthetic artemisinin: a model for the use of synthetic biology in pharmaceutical development. Nat Rev Microbiol.

[CR55] Ro D-K, Paradise EM, Ouellet M, Fisher KJ, Newman KL, Ndungu JM, Ho KA, Eachus RA, Ham TS, Kirby J (2006). Production of the antimalarial drug precursor artemisinic acid in engineered yeast. Nature.

[CR56] Rohdich F, Wungsintaweekul J, Fellermeier M, Sagner S, Herz S, Kis K, Eisenreich W, Bacher A, Zenk MH (1999) Cytidine 5′-triphosphate-dependent biosynthesis of isoprenoids: YgbP protein of *Escherichia coli* catalyzes the formation of 4-diphosphocytidyl-2-*C*-methylerythritol. Proc Natl Acad Sci 96(21):11758–11763 10.1073/pnas.96.21.11758 PMC18359 10518523

[CR57] Rohmer M (2008). From molecular fossils of bacterial hopanoids to the formation of isoprene units: discovery and elucidation of the methylerythritol phosphate pathway. Lipids.

[CR58] Rude MA, Schirmer A (2009). New microbial fuels: a biotech perspective. Curr Opin Microbiol.

[CR59] Sangari FJ, Perez-Gil J, Carretero-Paulet L, Garcia-Lobo JM, Rodriguez-Concepcion M (2010). A new family of enzymes catalyzing the first committed step of the methylerythritol 4-phosphate (MEP) pathway for isoprenoid biosynthesis in bacteria. Proc Natl Acad Sci U S A.

[CR60] Sato T, Yoshida S, Hoshino H, Tanno M, Nakajima M, Hoshino T (2011) Sesquarterpenes (C_35_ terpenes) biosynthesized via the cyclization of a linear C_35_ isoprenoid by a tetraprenyl-β-curcumene synthase and a tetraprenyl-β-curcumene cyclase: identification of a new terpene cyclase. J Am Chem Soc 133(25):9734–9737. doi:10.1021/ja203779h 10.1021/ja203779h 21627333

[CR61] Sauer U, Cameron DC, Bailey JE (1998). Metabolic capacity of *Bacillus subtilis* for the production of purine nucleosides, riboflavin, and folic acid. Biotechnol Bioeng.

[CR62] Schallmey M, Singh A, Ward OP (2004). Developments in the use of *Bacillus* species for industrial production. Can J Microbiol.

[CR63] Shirk MC, Wagner WP, Fall R (2002). Isoprene formation in *Bacillus subtilis*: a barometer of central carbon assimilation in a bioreactor?. Biotechnol Prog.

[CR64] Sivy TL, Shirk MC, Fall R (2002). Isoprene synthase activity parallels fluctuations of isoprene release during growth of *Bacillus subtilis*. Biochem Biophys Res Commun.

[CR65] Soga T, Ohashi Y, Ueno Y, Naraoka H, Tomita M, Nishioka T (2003). Quantitative metabolome analysis using capillary electrophoresis mass spectrometry. J Proteome Res.

[CR66] Soliman SS, Tsao R, Raizada MN (2011). Chemical inhibitors suggest endophytic fungal paclitaxel is derived from both mevalonate and non-mevalonate-like pathways. J Nat Prod.

[CR67] Sprenger GA, Schorken U, Wiegert T, Grolle S, de Graaf AA, Taylor SV, Begley TP, Bringer-Meyer S, Sahm H (1997). Identification of a thiamin-dependent synthase in *Escherichia coli* required for the formation of the 1-deoxy-D-xylulose 5-phosphate precursor to isoprenoids, thiamin, and pyridoxol. Proc Natl Acad Sci U S A.

[CR68] Stockton JR, Wyss O (1946). Proteinase production by *Bacillus subtilis*. J Bacteriol.

[CR69] Sun Z, Cunningham FX, Gantt E (1998). Differential expression of two isopentenyl pyrophosphate isomerases and enhanced carotenoid accumulation in a unicellular chlorophyte. Proc Natl Acad Sci U S A.

[CR70] Takagi M, Kaneda K, Shimizu T, Hayakawa Y, Seto H, Kuzuyama T (2004). *Bacillus subtilis ypgA* gene is *fni*, a nonessential gene encoding type 2 isopentenyl diphosphate isomerase. Biosci Biotechnol Biochem.

[CR71] Takahashi S, Kuzuyama T, Watanabe H, Seto H (1998) A 1-deoxy-D-xylulose 5-phosphate reductoisomerase catalyzing the formation of 2-*C*-methyl-D-elythritol 4-phosphate in an alternative nonmevalonate pathway for terpenoid biosynthesis. Proc Natl Acad Sci U S A 95(17):9879–9884. doi:10.1073/pnas.95.17.9879 10.1073/pnas.95.17.9879 PMC21430 9707569

[CR72] Thiele I, Palsson BØ (2010). A protocol for generating a high-quality genome-scale metabolic reconstruction. Nat Protoc.

[CR73] Tsuruta H, Paddon CJ, Eng D, Lenihan JR, Horning T, Anthony LC, Regentin R, Keasling JD, Renninger NS, Newman JD (2009). High-level production of amorpha-4, 11-diene, a precursor of the antimalarial agent artemisinin, in *Escherichia coli*. PLoS One.

[CR74] van der Werf MJ, Overkamp KM, Muilwijk B, Coulier L, Hankemeier T (2007). Microbial metabolomics: toward a platform with full metabolome coverage. Anal Biochem.

[CR75] Villas-Bôas SG, Bruheim P (2007). Cold glycerol–saline: the promising quenching solution for accurate intracellular metabolite analysis of microbial cells. Anal Biochem.

[CR76] Wagner WP, Helmig D, Fall R (2000). Isoprene biosynthesis in *Bacillus subtilis* via the methylerythritol phosphate pathway. J Nat Prod.

[CR77] Wagner WP, Nemecek-Marshall M, Fall R (1999). Three distinct phases of isoprene formation during growth and sporulation of *Bacillus subtilis*. J Bacteriol.

[CR78] Wang W, Oldfield E (2014). Bioorganometallic chemistry with IspG and IspH: structure, function, and inhibition of the Fe_4_S_4_ proteins involved in isoprenoid biosynthesis. Angew Chem Int Ed.

[CR79] Wang W, Wang K, Liu Y-L, No J-H, Li J, Nilges MJ, Oldfield E (2010). Bioorganometallic mechanism of action, and inhibition, of IspH. Proc Natl Acad Sci U S A.

[CR80] Westfall PJ, Pitera DJ, Lenihan JR, Eng D, Woolard FX, Regentin R, Horning T, Tsuruta H, Melis DJ, Owens A (2012). Production of amorphadiene in yeast, and its conversion to dihydroartemisinic acid, precursor to the antimalarial agent artemisinin. Proc Natl Acad Sci.

[CR81] Westers L, Westers H, Quax WJ (2004). *Bacillus subtilis* as cell factory for pharmaceutical proteins: a biotechnological approach to optimize the host organism. Bioinorg Chem Appl.

[CR82] Whited GM, Feher FJ, Benko DA, Cervin MA, Chotani GK, McAuliffe JC, LaDuca RJ, Ben-Shoshan EA, Sanford KJ (2010). Technology update: development of a gas-phase bioprocess for isoprene-monomer production using metabolic pathway engineering. Ind Biotechnol.

[CR83] Widner B, Behr R, Von Dollen S, Tang M, Heu T, Sloma A, Sternberg D, DeAngelis PL, Weigel PH, Brown S (2005). Hyaluronic acid production in *Bacillus subtilis*. Appl Environ Microbiol.

[CR84] Wilson RM, Danishefsky SJ (2006). Applications of total synthesis to problems in neurodegeneration: fascinating chemistry along the way. Acc Chem Res.

[CR85] Withers ST, Keasling JD (2007). Biosynthesis and engineering of isoprenoid small molecules. Appl Microbiol Biotechnol.

[CR86] Xue D, Abdallah II, de Haan IE, Sibbald MJ, Quax WJ (2015). Enhanced C_30_ carotenoid production in *Bacillus subtilis* by systematic overexpression of MEP pathway genes. Appl Microbiol Biotechnol.

[CR87] Xue J, Ahring BK (2011) Enhancing isoprene production by genetic modification of the 1-deoxy-D-xylulose-5-phosphate pathway in *Bacillus subtilis*. Appl Environ Microbiol 77(7):2399–2405. doi:10.1128/aem.02341-10 10.1128/AEM.02341-10 PMC3067423 21296950

[CR88] Yoshida K, Ueda S, Maeda I (2009). Carotenoid production in *Bacillus subtilis* achieved by metabolic engineering. Biotechnol Lett.

[CR89] Yuan LZ, Rouviere PE, Larossa RA, Suh W (2006). Chromosomal promoter replacement of the isoprenoid pathway for enhancing carotenoid production in *E. coli*. Metab Eng.

[CR90] Zhao L, Chang WC, Xiao Y, Liu HW, Liu P (2013). Methylerythritol phosphate pathway of isoprenoid biosynthesis. Annu Rev Biochem.

[CR91] Zhao Y, Yang J, Qin B, Li Y, Sun Y, Su S, Xian M (2011). Biosynthesis of isoprene in *Escherichia coli* via methylerythritol phosphate (MEP) pathway. Appl Microbiol Biotechnol.

[CR92] Zhou K, Zou R, Stephanopoulos G, Too H-P (2012). Metabolite profiling identified methylerythritol cyclodiphosphate efflux as a limiting step in microbial isoprenoid production. PLoS One.

[CR93] Zhou K, Zou R, Zhang C, Stephanopoulos G, Too HP (2013). Optimization of amorphadiene synthesis in *Bacillus subtilis* via transcriptional, translational, and media modulation. Biotechnol Bioeng.

[CR94] Zhou Y, Nambou K, Wei L, Cao J, Imanaka T, Hua Q (2013). Lycopene production in recombinant strains of *Escherichia coli* is improved by knockout of the central carbon metabolism gene coding for glucose-6-phosphate dehydrogenase. Biotechnol Lett.

